# Internal consistency of a synthetic population construction method for chronic disease micro-simulation models

**DOI:** 10.1371/journal.pone.0205225

**Published:** 2018-11-15

**Authors:** René Kooiker, Hendriek C. Boshuizen

**Affiliations:** 1 National Institute for Public Health and the Environment, Bilthoven, the Netherlands; 2 Wageningen University, Wageningen, the Netherlands; Sciensano, BELGIUM

## Abstract

**Background:**

Micro-simulation models of risk-factors and chronic diseases are built increasingly often, and each model starts with an initial population. Constructing such populations when no survey data covering all variables are available is no trivial task, often requiring complex methods based on several (untested) assumptions. In this paper, we propose a method for evaluating the merits of construction methods, and apply this to one specific method: the construction method used in the DYNAMO-HIA model.

**Methods:**

The initial population constructed using the DYNAMO-HIA method is compared to another population constructed by starting a simulation with only newborns and simulating the course taken by one risk-factor and several diseases. In this simulation, the age- and sex-specific prevalence of the risk-factor is kept constant over time.

**Results:**

Our simulations show that, in general, the DYNAMO-HIA method clearly outperforms a method that assumes independence of the risk-factor and the prevalence of diseases and independence between all diseases. In many situations the DYNAMO-HIA method performs reasonably well, but in some the proportion with the risk-factor for those with a disease is under- or overestimated by as much as 10 percentage points. For determining comorbidity between diseases linked by a common causal disease or a common risk-factor it also performs reasonably well. However, the current method performs poorly for determining the comorbidity between one disease caused by the other.

**Conclusion:**

The DYNAMO-HIA methods perform reasonably well; they outperform a baseline assumption of independence between the risk-factor and diseases in the initial population. The method for determining the comorbidity between diseases that are causally linked needs improvement. Given the existing discrepancies for situations with high relative risks, however, developing more elaborate methods based on running simulation models to generate an initial population would be worthwhile.

## Introduction

Demographic population health models can be used to model the effects of risk-factors on the transitions between health states (e.g. being healthy, diseased, or disabled), on their respective prevalence rates and durations, and on the population measures these health states affect, such as the life expectancy, structure, and size of the population. These models can be applied in Health Impact Assessment (HIA) in order to support decision-making in health policy, since they quantify population outcomes of policy interventions that influence risk-factor prevalence (e.g. the proportion of smokers) or transition rates between risk-factor states (e.g. start/stop rates in smoking).

Markov-models provide a widely used framework for such modeling needs, but would require an unworkable amount of states within the model for a realistic number of risk-factor states and diseases to be included. For instance, the probability of mortality and diseases can depend on exposure time. Thus, the number of states in a model would increase exponentially if it includes all relevant exposure history.

In such cases, micro-simulation or partial micro-simulation models [1] are more efficient. Micro-simulations model the lives of a large number of individuals over time, letting them acquire diseases (or not) until the end of life. Altering the risk-factors to which individuals are exposed in a simulation allows researchers to estimate the effects of interventions and health policies.

Examples of such models include the POHEM model ([2–5], the UK-Health Forum micro-simulation model [6–9], the OECD model for obesity [10], the US IMPACT Food Policy Model [11].

Such models not only require a quantitative description of the processes governing disease occurrence and mortality over time, but also an initial population that adequately represents the joint distribution of risk-factor states and diseases[12]. This initial population is otherwise known as the “static component of the model,” because it determines the value of variables before model time is "turned on"[10].

In some cases, survey data might exist for a population similar to the model's target population, and if these data also contain all information on risk-factors and diseases used in the model, then the initial population can simply be obtained from the individuals in the survey. If the simulation requires more individuals than are available in the survey, bootstrapping or other resampling methods can increase the population size. Weighting, then, can be applied for assuring that the resulting population is representative of the target population.

In other cases, however, there does not exist a single survey that covers all risk-factors and diseases needed for the simulation. In this case, a synthetic population needs to be made based on data from different sources. The advantage of this approach is that publicly available data sources can be used, and access to privacy-protected micro-data is not needed. Constructing such an initial population is no trivial task. Unfortunately, technical reports on micro-simulation models focus mostly on the estimation and calibration of the transition rates of the model, and on the way these are used during simulation. More often than not, authors omit an adequate description of ways to actually construct initial populations. For instance, a paper on the EConDA model[13] does not describe the construction of the initial state of each simulated individual, while Sassi et al.[10] only described their method for assigning risk-factor states, not for disease states. Neither paper specifies whether their models assign a different probability of disease to individuals with a different risk-factor state, and if they do, which method is used.

In the partial micro-simulation model DYNAMO-HIA (Dynamic Modeling for Health Impact Assessment)[1], an initial population is constructed from marginal data (available as population statistics) based on the assumption that the prevalence odds ratio (POR) of disease between different risk-factor states is equal to the incidence rate ratio (RR) for those risk-factor states as observed in epidemiological studies. Similarly, it is assumed that the prevalence odds ratio of a disease that is caused by another disease–within the same risk-factor stratum–is equal to the RR of that disease on the risk of the disease under consideration. Boshuizen et al. [1] considered this assumption to be justified because under certain conditions, the POR of a disease equals the RR. Neil Pearce has outlined the conditions and the reasoning underlying the assumption [14]. He presents four conditions under which the POR estimates the RR: 1) the population is in a steady state (stationary) over time (in that the numbers within each subpopulation defined by exposure, disease, and covariates do not change with time); 2) incidence rates and exposure and disease status are unrelated to the immigration and emigration rates and population size; 3) average disease duration does not change over time; 4) the average duration of disease is the same in the exposed and non-exposed groups (i.e., exposure has no effect on mortality from the disease or recovery rates).

In the DYNAMO-HIA model, the second and third assumptions also apply to the simulation of disease prevalence over time. However, this is not the case for the fourth assumption, since overall mortality rates in the model (and thus disease duration) can vary according to risk-factor status. Furthermore, an important implicit assumption in Pearce’s four conditions seems to be that relative risks of exposure on incidence are independent of age. This need not be the case in DYNAMO-HIA simulation, since the model can use age-specific data on those relative risks. A more accurate representation of reality, perhaps, but it might affect the accuracy of the initial population constructed with it.

For all these reasons, it is quite possible that significant assumptions underlying the calculation of the initial population in DYNAMO-HIA are not consistent with assumptions made during simulation (specifically, assumptions that justify approximating the POR to the IRR). In this article, therefore, we propose a method for assessing how well an initial population construction performs its task. This involves estimating to what extent and under which circumstances the method for constructing an initial population delivers a population that is consistent under simulation. We intend 'consistency' under simulation to mean that the constructed initial population is similar to the population as it would emerge from simulation de novo, that is, from a population of newborns free of disease. We will illustrate the method by testing the initial population module from DYNAMO-HIA.

## Methods

**Fig 1:**
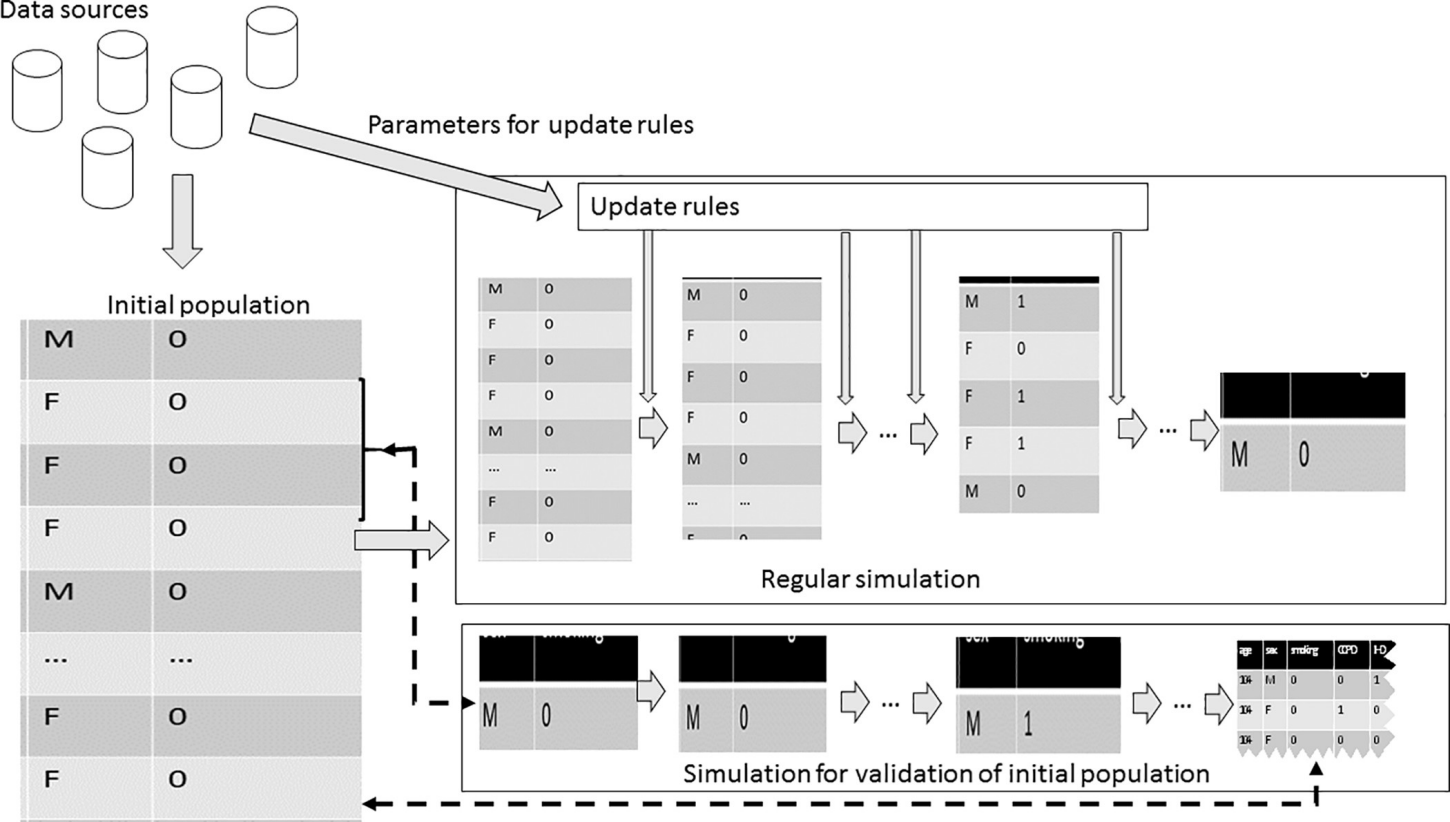
Schematic view of the proposed validation methodology for initial population construction.

Fig 1 gives a schematic overview of the modeling process and the proposed validation method for the initial population.

In this figure, te initial population as constructed for regular simulation is compared to the population as it emerges from a simulation starting only with a cohort of new-borns.

Micro-simulation models a population's development over time. Each individual is assigned specific characteristics, such as age, sex, smoking status, and disease status. With each time interval, the model updates these characteristics according to specific rules. In DYNAMO-HIA, the update rule is as follows: for each risk-factor, transition probabilities are used to randomly draw a new risk-factor state. For disease states, the model updates the probability of having the disease, based on age, sex, risk-factor and other disease status. Lastly, DYNAMO-HIA updates each individual's probability of survival using the mortality rate, which depends on age, sex, disease status and optionally risk-factor status. Appendix A describes these update rules used in DYNAMO-HIA in more detail. External data sources provide input for constructing the initial population for the simulation and for the parameters of the update rules. Some sources (such as the RRs of the risk-factor on a disease) provide data for both the initial population construction and the parameters, while other sources are only used for one of these (e.g. the population incidence rate of a disease is only used to calculate parameters of the update rule for that disease).

In order to assess the extent to which the assumptions behind an initial population construction are consistent with the population as it emerges from simulation, we compare a cohort of newborns simulated up to old age to an initial population. For the cohort of newborns, we project risk-factor biographies using specific transition probabilities that keep the age- and sex-specific risk-factor probabilities equal to that in the examined initial population. Subsequently, we calculate disease probabilities based on the risk-factor biographies. In case of chronic diseases, newborns are mostly born disease-free, so we do not use disease prevalence rates directly from input data. Instead, disease prevalence rates emerge by applying incidence and mortality rates over the course of years of simulation. Thus, a cohort of newborns comes to represent a population at 'equilibrium' (under the current risk-factor exposure, and constant incidence and mortality rates), which we interpret as a ‘history’ of the initial population.

One could also construct a set of incidence and disease-related excess mortality rates that would result in an age-specific disease prevalence equal to the current disease prevalence. However, the aim of modeling is to model dynamic situations, so we use realistic incidence and excess mortality rates instead. This implies that the age-specific prevalence of disease in the ‘equilibrium’ population (based on the newborn cohort) differs from the age-specific prevalence of disease in the initial population.

### Evaluation criteria

To evaluate the method of initial population construction as described above, we examine two factors. First, whether the joint distribution of the risk-factor and each disease is consistent with the distribution resulting from simulation in a birth cohort. We do so by looking at the distribution of the risk-factors in individuals who have the corresponding disease. Since the age-specific risk-factor distribution in the total population remains constant over time, the age-specific risk-factor distribution in those *with* the disease should also remain constant over time. Thus, if the assumptions behind the initial population module hold to a reasonable extent, the resulting simulated (future) risk-factor distribution within disease categories of this newborn cohort should resemble those rates in the initial population.

The second factor is the validity of the joint distribution of diseases. In our simulations the age-specific prevalence of each disease over time is not constant, so the prevalence of one disease for those having another disease should not be constant either. Therefore, we look at comorbidity ratios instead of the method used to evaluate the joint distribution with risk-factors (described above). The comorbidity ratio of two disease A and B is defined as:

$$Comorbidity\;ratio=\frac{P_{AB}}{P_{A}P_{B}}$$

were *P*_*A*_ is the prevalence of disease A, *P*_*B*_ is the prevalence of disease B and *P*_*AB*_ is the prevalence of having both disease A and disease B. We calculate the comorbidity ratio separately for each combination of age and sex.

In our case, if the difference in prevalence rates over time is relatively small, the comorbidity ratio should not be affected. Comparing the comorbidity ratios between the initial and steady state population then tells us whether the method for construction of the initial population is valid.

### Application to validating the initial population module of DYNAMO-HIA

DYNAMO-HIA constructs an initial population from marginal data: risk-factor prevalence and disease prevalence. 'Marginal' means that we only use data on, say, the percentage of smokers, or the percentage of the population with lung cancer, specified by age and sex. No data on the percentage of smokers in individuals with lung cancer is necessary. The initial population module then calculates the risk-factor-specific prevalence of disease using the assumption that the PORs are well approximated by the RRs as observed in epidemiological studies [15]. Similarly, it calculates the amount of comorbidity assuming that the POR of a disease caused by another disease–within the same risk-factor stratum–is equal to the RR of that second disease on the incidence of the first. Since the approximation of PORs by RRs is also conditional on the presence of other risk-factors like age and sex [16], the initial population module calculates the joint distribution for each age and sex separately using input data for each age and sex.

DYNAMO-HIA derives its methods for constructing the initial population from similar methods used in the RIVM-CDM model [17]. In Appendix B we describe the construction method in more detail.

This way of constructing an initial population from marginal data differs from approaches based on “calibration” or “alignment”. These assume that a population sample (micro-data) is available that already contains all variables (including their correlations), but lacks the correct marginal distribution. In this situation, different alignment and calibration methods are available to construct a population with the correct marginal distribution.

#### Constructing simulation runs

Our method prescribes the use of stationary risk-factors; that is, the age- and sex-specific risk-factor distribution over time in the newborn simulation should equal the corresponding distribution in the initial population. DYNAMO-HIA offers the option of enforcing stationary risk-factors by applying so-called net-transition rates. In this mode, DYNAMO-HIA internally estimates the transition probabilities that keep the age- and sex-specific risk-factor prevalence constant. We used this option for our simulation runs.

When running simulations with many different risk-factors—such as alcohol, cholesterol levels, and fruit consumption—smoking often showed the greatest differences between the newborn and the initial cohorts. Perhaps this is unsurprising, since smoking is a risk-factor that has comparatively high relative risks on mortality and incidence. Instead of presenting many simulations with different risk-factors, we chose a worst-case scenario approach. We therefore chose to present results for smoking along with one other risk-factor. For this we chose BMI, a risk-factor with a strong effect on diabetes. Another reason to take BMI is that its development is also (but not uniquely) influenced by the physiology of aging. For smoking, a behavioral factor, this is not the case; it instead tracks the influence of cultural and generational changes.

For our worst-case scenario, we used smoking as a risk-factor in a simulation containing the following diseases: diabetes, coronary heart disease (CHD), stroke, congestive heart failure, and chronic obstructive pulmonary disease (COPD), lung cancer, oral cavity cancer, and larynx cancer.

For our BMI scenario, we used the following diseases: diabetes, coronary heart disease (CHD), stroke, congestive heart failure, breast cancer, colorectal cancer, prostate cancer, kidney cancer and endometrial cancer. In all cases diabetes was considered a causal disease for CHD, stroke, and heart failure. We assumed no effect of smoking on diabetes. All data were estimates for the Dutch population in 2011. We used 2000 simulated individuals in each one-year age and sex group for the risk factor simulations, and 500 for those looking at comorbidity.

### Statistical analysis

Statistical analyses were carried out in two stages of the work: in preparing input data for the DYNAMO-HIA model from the raw data sources, and in analyzing the results from the simulation. The simulation itself was carried out on DYNAMO-HIA version 2.08 (www.dynamo-hia.eu).

#### Preparing the input data for DYNAMO-HIA

Cancer incidence rates (1989–2011) and survival data (1989–2010) were available from the Netherlands Cancer Registry. Poisson models (including time-trends) were used to model age- and sex-specific excess mortality and incidence rates in 2011. Stroke, diabetes (type 2), congestive heart failure, and coronary heart disease (CHD) incidence rates were taken from the Netherlands Information Network of General Practice (LINH) for 2011. For stroke and CHD, patients were linked to hospital records (1995–2010) in order to obtain only incident cases in those without previously registered cases of CHD and stroke respectively, and were also linked to cause-of-death records from the Dutch national mortality register in order to include acutely fatal cases. Both prevalent and incident cases were linked to death records in order to obtain excess mortality. For these two diseases, mortality was split into short term (less than 1 year) and long term mortality. Acute mortality was calculated by subtracting long-term mortality from the observed one-year mortality. COPD and diabetes incidence data came from the same GP registration. Again, both prevalent and incident cases were linked to death records in order to determine excess mortality rates. Because of the scarceness of disease data at high ages, incidence, prevalence and disease related excess mortality above age 87 were kept equal to that at age 87. All data were differentiated by age and sex. Analyses were carried out in the software R.

We used smoking prevalence rates as obtained from a 2011 smoking survey conducted by the Dutch Foundation on Smoking and Health in the population aged 16 and over. Smoking data comprised the proportion of current smokers, former smokers and non-smokers. Smoking rates were assumed zero until and including age 10. From that point on we let the rates increase linearly with age toward the observed prevalence rates at age 16. We used a multinomial spline model (package VGAM in R) to smooth the smoking rates over age.

Our relative risk rates came from previous chronic disease modeling in the Netherlands, which smoothly vary with age. They are given in the supplementary material (S1–S3 Figs) For cardiovascular diseases they decrease with age, because equivalent absolute risk differences translate to a lower relative risk when the baseline risk (that is, the risk in non-smokers) increases with age. For COPD and lung cancer, the relative risks first increase with age, reflecting higher average cumulative exposure with age; at older age, however, the effect of increasing baseline risk takes over and relative risks decline with age [18].

#### Analysis of the results of the simulation

In each risk-factor category (e.g., never, current, and former smokers) the proportion of individuals with a particular disease as well as comorbidity ratios were plotted against age for each sex, both for the initial population (“initial”) or the newborn cohort (“newborns”). Differences at particular ages were calculated and tabled. Running the model one hundred times with different random seeds provided the 95% confidence intervals, estimating the amount of uncertainty due to Monte Carlo error.

## Results

**Fig 2:**
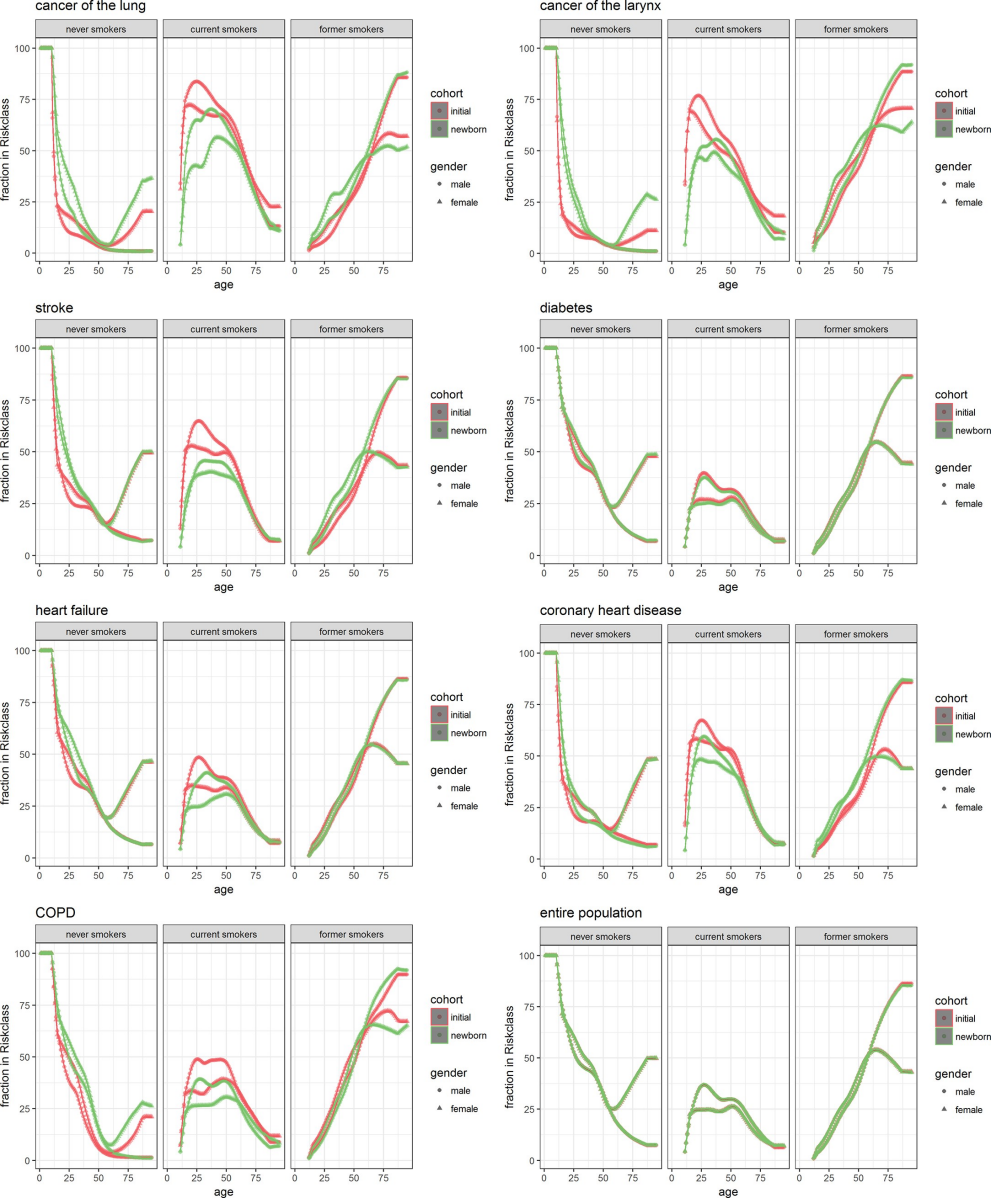
**Prevalence of disease by age and sex within different strata of smoking**, Separately for the initial population as constructed for DYNAMO-HIA (red) and from simulation of a cohort of new-borns (green) for men(dots) and women (triangles).

Fig 2 shows the proportion in each risk-factor category (never, current, and former smokers) in the entire population (bottom right) and in populations with a particular disease (other plots).

The different panels in the subplots indicate each risk class. They are to be interpreted as follows: of the people with the disease, the y-axis denotes the proportion of people in that risk class. That is, a y-value of 0.25 at age 50 for lung cancer on the curve for women in the initial cohort would mean that 25% of women with lung cancer from the initial cohort are in the never smokers category. The sum of the corresponding points on the other curves for each risk class is 1. Thus, combining the panels for a particular disease represents how the people with the disease are distributed across risk classes. In this plot, we want to look for differences between the initial and newborn cohort. For the newborn cohort we also plotted 95% confidence intervals reflecting the simulation error. However, this error is too small to be visible in the plots.

Looking at Fig 2, we see that there is only a small difference in smoking proportions between the cohorts in the entire population. The expectation is that both cohorts would have exactly the same proportions of smoking, as we used transition rates aimed to achieve this end. Table 1 shows the difference between the lines for 4 ages with 95% confidence intervals reflecting the Monte Carlo simulation error. It shows that only for men at high ages simulation error cannot fully explain the differences. This small difference is most likely because the prevalence of disease in the newborn cohort at high ages is different from that in the initial cohort. This results in a different selective mortality. The plot for diabetes—which in this simulation was not influenced by smoking—is quite similar. For the other diseases, we see the largest differences mostly in the proportion of current smokers and at the ages below 50. Differences below the age of 50 are not very influential in modeling because there is no significant prevalence of any of the simulated diseases below this age.

**Table 1:** Difference in the proportion in different smoking categories between the initial population (approximate method) and the newborn cohort in percent points, based on 100 simulations with 95% confidence intervals^1^.

age	disease	never smokers	current smokers	former smokers	never smokers	current smokers	former smokers
50	entire population	0.02 [-0.19; 0.22 ]	0.02 [-0.18; 0.22 ]	-0.03 [-0.23; 0.17 ]	-0.05 [-0.24; 0.14 ]	0.05 [-0.13; 0.23 ]	0.00 [-0.19; 0.19 ]
50	with cancer of the lung	-0.61 [-0.64; -0.57 ]	6.23 [5.99; 6.48 ]	-5.63 [-5.86; -5.39 ]	-1.07 [-1.12; -1.02 ]	10.74 [10.48; 10.99 ]	-9.66 [-9.9; -9.42 ]
50	with cancer of the oral cavity	-1.09 [-1.18; -1 ]	8.51 [8.25; 8.77 ]	-7.42 [-7.65; -7.19 ]	-1.66 [-1.75; -1.58 ]	11.99 [11.75; 12.24 ]	-10.33 [-10.56; -10.1 ]
50	with cancer of the larynx	-0.48 [-0.53; -0.43 ]	5.72 [5.45; 5.98 ]	-5.24 [-5.49; -4.99 ]	-0.41 [-0.45; -0.36 ]	6.9 [6.66; 7.14 ]	-6.49 [-6.73; -6.25 ]
50	with stroke	-0.43 [-0.59; -0.28 ]	8.32 [8.07; 8.57 ]	-7.89 [-8.1; -7.68 ]	-0.31 [-0.44; -0.18 ]	11.29 [11.06; 11.52 ]	-10.98 [-11.19; -10.76 ]
50	with diabetes	-0.49 [-0.69; -0.29 ]	0.99 [0.78; 1.19 ]	-0.50 [-0.7; -0.3 ]	-0.66 [-0.85; -0.47 ]	1.21 [1.03; 1.39 ]	-0.55 [-0.74; -0.36 ]
50	with heart failure	-0.27 [-0.45; -0.1 ]	2.29 [2.07; 2.52 ]	-2.02 [-2.23; -1.82 ]	-0.84 [-1; -0.67 ]	3.24 [3.04; 3.44 ]	-2.40 [-2.6; -2.2 ]
50	with coronary heart disease	1.64 [1.52; 1.76 ]	6.17 [5.91; 6.43 ]	-7.81 [-8.04; -7.58 ]	0.21 [0.09; 0.33 ]	10.94 [10.7; 11.18 ]	-11.15 [-11.37; -10.93 ]
50	with COPD	-8.83 [-8.95; -8.71 ]	9.05 [8.81; 9.29 ]	-0.22 [-0.45; 0 ]	-8.45 [-8.57; -8.34 ]	8.37 [8.17; 8.57 ]	0.08 [-0.12; 0.29 ]
**age**	**disease**	**never smokers**	**current smokers**	**former smokers**	**never smokers**	**current smokers**	**former smokers**
65	entire population	-0.06 [-0.24; 0.13 ]	-0.06 [-0.24; 0.12 ]	0.12 [-0.09; 0.32 ]	-0.08 [-0.28; 0.11 ]	-0.10 [-0.24; 0.05 ]	0.18 [-0.02; 0.38 ]
65	with cancer of the lung	-0.03 [-0.05; -0.01 ]	5.69 [5.41; 5.97 ]	-5.66 [-5.94; -5.39 ]	-4.49 [-4.63; -4.36 ]	4.35 [4.1; 4.61 ]	0.14 [-0.09; 0.37 ]
65	with cancer of the oral cavity	0.17 [0.1; 0.23 ]	8.82 [8.56; 9.08 ]	-8.99 [-9.24; -8.74 ]	-3.58 [-3.72; -3.43 ]	5.55 [5.32; 5.79 ]	-1.98 [-2.2; -1.76 ]
65	with cancer of the larynx	-0.10 [-0.13; -0.07 ]	5.89 [5.66; 6.12 ]	-5.79 [-6.02; -5.57 ]	-3.74 [-3.85; -3.63 ]	2.94 [2.72; 3.15 ]	0.80 [0.59; 1.01 ]
65	with stroke	1.26 [1.14; 1.39 ]	4.64 [4.41; 4.86 ]	-5.90 [-6.11; -5.68 ]	0.50 [0.34; 0.67 ]	1.44 [1.24; 1.65 ]	-1.95 [-2.15; -1.74 ]
65	with diabetes	-0.16 [-0.34; 0.01 ]	0.72 [0.54; 0.91 ]	-0.56 [-0.76; -0.36 ]	-0.36 [-0.55; -0.18 ]	0.34 [0.19; 0.48 ]	0.03 [-0.17; 0.22 ]
65	with heart failure	0.17 [0.02; 0.31 ]	1.81 [1.61; 2.01 ]	-1.97 [-2.18; -1.77 ]	-0.49 [-0.66; -0.32 ]	0.43 [0.26; 0.59 ]	0.07 [-0.13; 0.26 ]
65	with coronary heart disease	2.16 [2.04; 2.28 ]	2.23 [2.01; 2.45 ]	-4.39 [-4.6; -4.18 ]	0.55 [0.39; 0.72 ]	-0.69 [-0.91; -0.48 ]	0.14 [-0.07; 0.35 ]
65	with COPD	-1.39 [-1.43; -1.35 ]	6.07 [5.88; 6.27 ]	-4.69 [-4.88; -4.49 ]	-5.48 [-5.59; -5.38 ]	4.45 [4.26; 4.64 ]	1.03 [0.84; 1.22 ]
**age**	**disease**	**never smokers**	**current smokers**	**former smokers**	**never smokers**	**current smokers**	**former smokers**
75	entire population	-0.12 [-0.29; 0.04 ]	-0.21 [-0.36; -0.07 ]	0.34 [0.13; 0.55 ]	-0.07 [-0.28; 0.14 ]	0.00 [-0.14; 0.13 ]	0.07 [-0.13; 0.28 ]
75	with cancer of the lung	0.06 [0.04; 0.08 ]	2.98 [2.7; 3.25 ]	-3.04 [-3.31; -2.77 ]	-11.08 [-11.29; -10.87 ]	5.61 [5.31; 5.9 ]	5.47 [5.24; 5.71 ]
75	with cancer of the oral cavity	0.50 [0.44; 0.56 ]	6.26 [6.04; 6.49 ]	-6.76 [-6.99; -6.53 ]	-9.57 [-9.79; -9.35 ]	5.62 [5.36; 5.87 ]	3.95 [3.73; 4.17 ]
75	with cancer of the larynx	-0.05 [-0.08; -0.03 ]	4.63 [4.44; 4.82 ]	-4.57 [-4.76; -4.38 ]	-11.79 [-11.97; -11.61 ]	4.64 [4.42; 4.87 ]	7.14 [6.93; 7.36 ]
75	with stroke	1.21 [1.08; 1.34 ]	1.29 [1.11; 1.47 ]	-2.5 [-2.72; -2.29 ]	-0.48 [-0.7; -0.26 ]	-0.74 [-0.94; -0.54 ]	1.22 [1.01; 1.42 ]
75	with diabetes	-0.18 [-0.34; -0.02 ]	0.37 [0.22; 0.51 ]	-0.18 [-0.39; 0.03 ]	-0.56 [-0.77; -0.35 ]	0.29 [0.15; 0.43 ]	0.27 [0.07; 0.48 ]
75	with heart failure	0.16 [0.02; 0.3 ]	0.53 [0.36; 0.69 ]	-0.68 [-0.89; -0.48 ]	-0.98 [-1.19; -0.78 ]	0.29 [0.12; 0.45 ]	0.69 [0.49; 0.9 ]
75	with coronary heart disease	1.58 [1.46; 1.7 ]	0.6 [0.44; 0.77 ]	-2.18 [-2.38; -1.98 ]	-2.12 [-2.34; -1.91 ]	-1.58 [-1.78; -1.38 ]	3.7 [3.5; 3.91 ]
75	with COPD	-0.32 [-0.35; -0.29 ]	6.76 [6.61; 6.92 ]	-6.45 [-6.61; -6.29 ]	-10.47 [-10.63; -10.3 ]	3.11 [2.91; 3.31 ]	7.35 [7.15; 7.55 ]
**age**	**disease**	**never smokers**	**current smokers**	**former smokers**	**never smokers**	**current smokers**	**former smokers**
85	entire population	-0.27 [-0.42; -0.12 ]	-0.44 [-0.55; -0.33 ]	0.71 [0.52; 0.89 ]	0.03 [-0.19; 0.24 ]	-0.09 [-0.19; 0.02 ]	0.06 [-0.14; 0.26 ]
85	with cancer of the lung	0.10 [0.08; 0.11 ]	1.27 [1.09; 1.46 ]	-1.37 [-1.56; -1.18 ]	-14.23 [-14.45; -14.01 ]	7.51 [7.3; 7.72 ]	6.72 [6.52; 6.92 ]
85	with cancer of the oral cavity	0.68 [0.62; 0.74 ]	4.25 [4.11; 4.38 ]	-4.93 [-5.08; -4.78 ]	-11.53 [-11.76; -11.3 ]	5.76 [5.59; 5.93 ]	5.77 [5.58; 5.97 ]
85	with cancer of the larynx	-0.07 [-0.09; -0.04 ]	3.51 [3.39; 3.63 ]	-3.45 [-3.57; -3.33 ]	-17.08 [-17.27; -16.88 ]	6.04 [5.87; 6.21 ]	11.04 [10.84; 11.23 ]
85	with stroke	0.60 [0.46; 0.73 ]	-0.42 [-0.54; -0.3 ]	-0.18 [-0.35; 0 ]	-0.35 [-0.57; -0.13 ]	-1.05 [-1.17; -0.93 ]	1.41 [1.21; 1.6 ]
85	with diabetes	-0.36 [-0.51; -0.22 ]	0.02 [-0.09; 0.13 ]	0.34 [0.17; 0.52 ]	-0.6 [-0.82; -0.38 ]	0.21 [0.1; 0.32 ]	0.39 [0.19; 0.59 ]
85	with heart failure	-0.02 [-0.15; 0.11 ]	-0.21 [-0.33; -0.09 ]	0.23 [0.06; 0.4 ]	-0.13 [-0.35; 0.08 ]	0.07 [-0.05; 0.2 ]	0.06 [-0.14; 0.26 ]
85	with coronary heart disease	1.05 [0.93; 1.16 ]	0.37 [0.26; 0.47 ]	-1.41 [-1.57; -1.25 ]	-0.14 [-0.36; 0.08 ]	-0.65 [-0.77; -0.52 ]	0.79 [0.59; 0.98 ]
85	with COPD	0.13 [0.11; 0.16 ]	3.23 [3.12; 3.33 ]	-3.36 [-3.47; -3.25 ]	-8.70 [-8.88; -8.52 ]	1.07 [0.92; 1.22 ]	7.63 [7.44; 7.82 ]

^1^Confidence intervals of the mean of the 100 simulations. Note that this is more accurate than the data presented in Figs 2, 3 and 4, which are based on a single simulation.

At all ages, but especially below the age of 50, the proportion in current smokers is much higher in the newborn cohort than in the initial population for the cancers and COPD, while for cardiovascular diseases this only holds for ages up to around 75 to 80. In the male cancer and COPD patients the decrease in the proportion of current smokers is compensated by an increase in the proportion of former smokers. In contrast, for female cancer and COPD patients the proportion of never smokers is higher in the newborn cohort. The latter, however, could be due to the use of net transition rates in combination with generational effects in smoking behavior of the Dutch population. Older generations of women are largely never smokers, while younger generations smoked in much larger proportions. As a result, age-specific proportions of smoking in women can only be kept constant by turning smokers into never smokers. This causes a flow of individuals with smoking-related diseases into the never smoking category, a flow that does not exist in real life. For the cardiovascular diseases above age 50 the differences between the newborn cohort and the initial population in the other smoking categories are small and mostly show an increase of the proportion of former smokers in the newborn cohort. The differences between the newborn cohort and initial population, however, are considerably smaller than the differences between smoking proportions in those with a smoking related disease and those in the general population.

Table 1 further shows that in some cases differences can be as high as 10 percent points, even reaching 15 percent points for 85 year old women who have never smoked.

**Fig 3:**
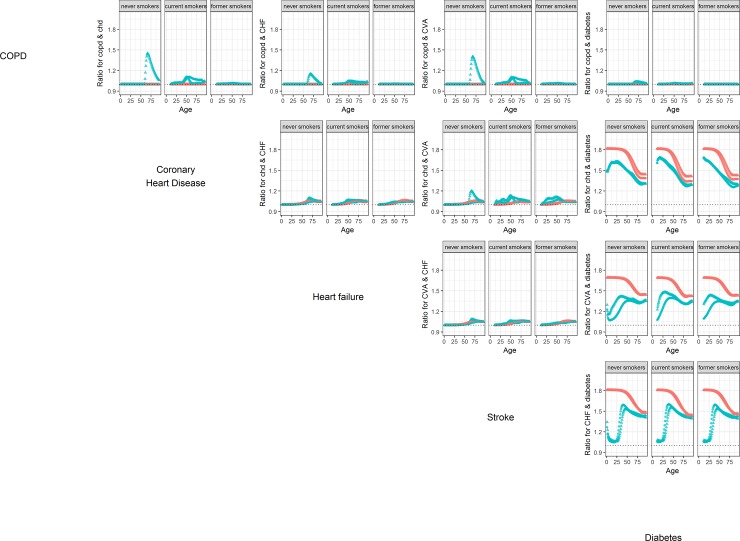
Comorbidity ratio’s by age and sex between all combinations of the 5 non-cancer diseases in the smoking simulation. Separately for the initial population as constructed for DYNAMO-HIA (red) and from the population as it emerges from simulation of a cohort of new-borns (green) and for men (dots) and women (triangles).

Fig 3 shows comorbidity ratios between different diseases within the smoking groups.

Here the simulation error is very small and therefore not visible on the plots. We do not show comorbidity relations with the cancers, as they are, like COPD, only linked to diabetes and cardiovascular disease via the relation with smoking, and thus show results similar to COPD. For COPD we expected comorbidity ratios with the other diseases to be equal to 1. However, in the newborn cohort individuals change smoking status during their lives. As described above, the net transition rates force some smoking women at higher ages into the category of never smokers. These women have a higher probability of both COPD and cardiovascular diseases, and an even higher increase in the probability of comorbidity. This explains the peak seen in the comorbidity ratio for older never smoking women.

For the comorbidity between CHD, stroke, and heart failure, which all depend on diabetes, we see that the comorbidity ratio increases slightly with age, both in the initial population and in the simulated newborn cohort. The comorbity ratios in both cohorts are rather similar, with the exception of the comorbidity between stroke and CHD.

The largest differences between newborn cohort and initial population occur in the comorbidities between the cardiovascular diseases and diabetes, which is modeled as a risk-factor for all these diseases. Here, we see that the comorbidity ratios in the initial population are much higher than those in the newborn cohort. A closer look at the method for initial population construction shows why this is the case. The method assume that the relative risk of diabetes on the disease is equal to the prevalence odds ratio. However, this relative risk is only applicable to individuals who contract diabetes first and the cardiovascular disease second. In the simulation, however, it is also possible to contract the cardiovascular disease first and diabetes second. Therefore, the simulated comorbidity is lower than the one assumed by the method.

**Fig 4:**
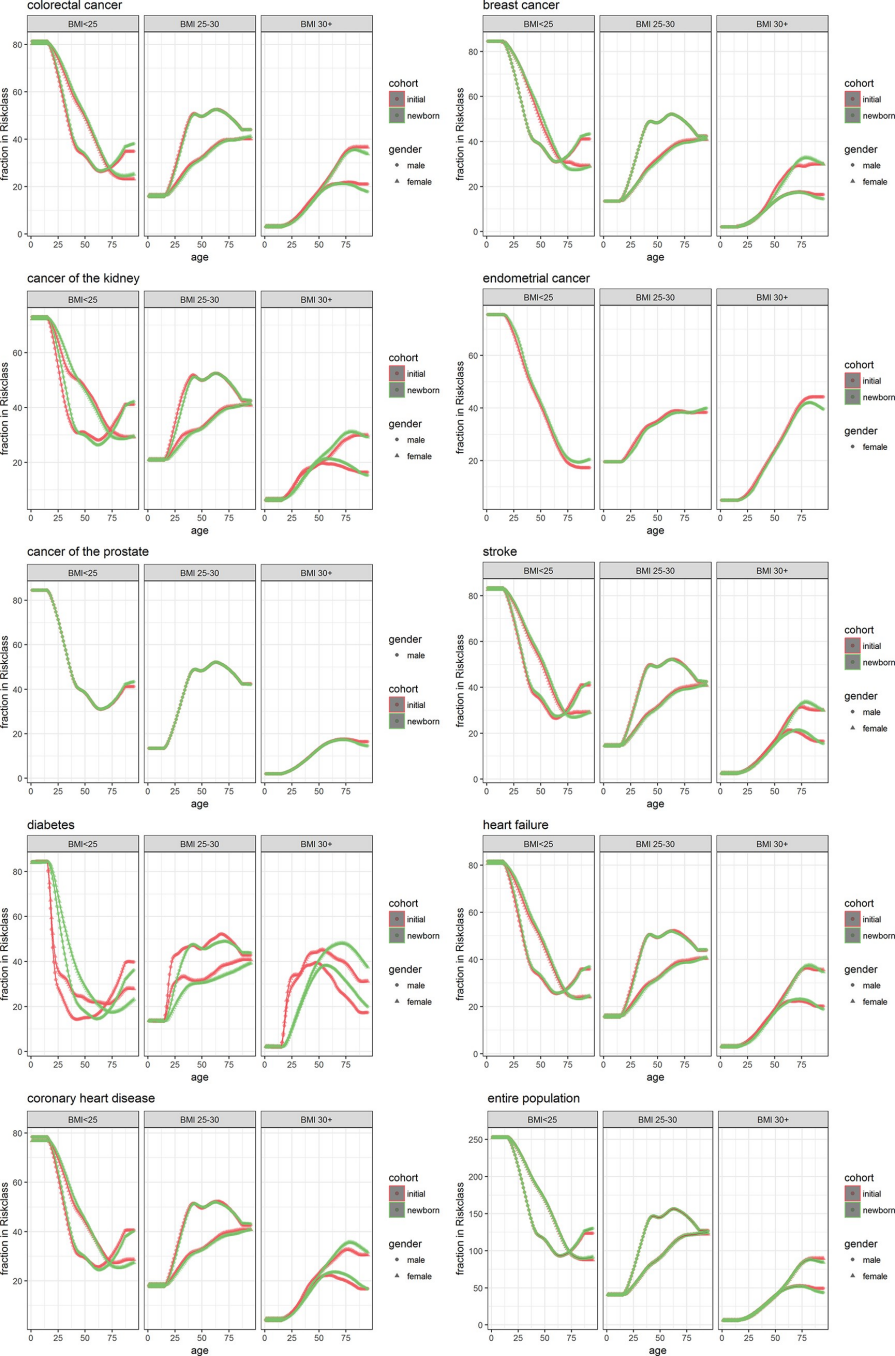
Prevalence of disease by age and sex within different strata of BMI. Separately for the initial population as constructed for DYNAMO-HIA (red) and from simulation of a cohort of new-borns (green) for men (dots) and women(triangles.

Fig 4 and Table 2 show the results for the simulation with BMI as risk-factor.

**Table 2:** Difference in the proportion in different categories of BMI between the initial population (approximate method) and the newborn cohort in percent points.

**age**	**disease**	**BMI < 25**	**25–30**	**30+**	**BMI < 25**	**25–30**	**30+**
50	entire population	-0.29 [-0.86; 0.28 ]	-0.06 [-0.64; 0.51 ]	0.35 [-0.05; 0.76 ]	-0.18 [-0.67; 0.32 ]	0.29 [-0.3; 0.87 ]	-0.11 [-0.49; 0.27 ]
50	with colorectal cancer	-0.63 [-0.81; -0.45 ]	-0.04 [-0.23; 0.16 ]	0.67 [0.51; 0.83 ]	-1.08 [-1.24; -0.92 ]	0.41 [0.21; 0.62 ]	0.67 [0.5; 0.83 ]
50	with breast cancer	-0.10 [-0.29; 0.09 ]	-0.02 [-0.21; 0.17 ]	0.12 [-0.02; 0.25 ]	-3.49 [-3.65; -3.32 ]	0.99 [0.79; 1.19 ]	2.49 [2.34; 2.65 ]
50	with cancer of the kidney	0.97 [0.81; 1.14 ]	-0.15 [-0.34; 0.05 ]	-0.83 [-1.01; -0.65 ]	1.01 [0.85; 1.16 ]	0.34 [0.13; 0.55 ]	-1.35 [-1.53; -1.17 ]
50	with endometrial cancer	-	-	-	-1.32 [-1.48; -1.17 ]	0.63 [0.41; 0.86 ]	0.69 [0.49; 0.9 ]
50	with cancer of the prostate	-0.10 [-0.29; 0.09 ]	-0.02 [-0.21; 0.17 ]	0.12 [-0.02; 0.25 ]	-	-	-
50	with stroke	-1.38 [-1.57; -1.2 ]	0.06 [-0.13; 0.25 ]	1.32 [1.17; 1.47 ]	-1.58 [-1.74; -1.42 ]	0.45 [0.25; 0.66 ]	1.12 [0.98; 1.27 ]
50	with diabetes	-2.70 [-2.82; -2.58 ]	0.01 [-0.22; 0.24 ]	2.7 [2.43; 2.96 ]	-5.21 [-5.34; -5.07 ]	1.13 [0.89; 1.37 ]	4.08 [3.8; 4.36 ]
50	with heart failure	-0.66 [-0.84; -0.48 ]	-0.18 [-0.38; 0.01 ]	0.84 [0.67; 1.01 ]	-1.18 [-1.34; -1.02 ]	0.35 [0.14; 0.55 ]	0.84 [0.67; 1 ]
50	with coronary heart disease	-0.36 [-0.53; -0.2 ]	-0.40 [-0.59; -0.2 ]	0.76 [0.57; 0.95 ]	-0.90 [-1.06; -0.74 ]	0.30 [0.09; 0.52 ]	0.59 [0.4; 0.79 ]
**age**	**disease**	**BMI < 25**	**25–30**	**30+**	**BMI < 25**	**25–30**	**30+**
65	entire population	-0.39 [-1; 0.21 ]	-0.05 [-0.73; 0.63 ]	0.44 [-0.01; 0.88 ]	-0.42 [-1.01; 0.17 ]	0.46 [-0.12; 1.05 ]	-0.05 [-0.5; 0.41 ]
65	with colorectal cancer	-0.24 [-0.42; -0.06 ]	0.14 [-0.09; 0.36 ]	0.11 [-0.07; 0.28 ]	-0.89 [-1.07; -0.71 ]	0.56 [0.37; 0.76 ]	0.33 [0.15; 0.51 ]
65	with breast cancer	-0.13 [-0.33; 0.07 ]	-0.02 [-0.24; 0.21 ]	0.15 [0; 0.29 ]	-1.31 [-1.49; -1.13 ]	0.64 [0.45; 0.83 ]	0.67 [0.49; 0.84 ]
65	with cancer of the kidney	1.74 [1.56; 1.92 ]	0.05 [-0.17; 0.27 ]	-1.79 [-1.97; -1.62 ]	1.64 [1.46; 1.83 ]	0.63 [0.44; 0.83 ]	-2.28 [-2.45; -2.1 ]
65	with endometrial cancer	-	-	-	-1.81 [-1.97; -1.65 ]	0.77 [0.58; 0.97 ]	1.04 [0.84; 1.24 ]
65	with cancer of the prostate	-0.13 [-0.33; 0.07 ]	-0.02 [-0.24; 0.21 ]	0.15 [0; 0.29 ]	-	-	-
65	with stroke	-0.90 [-1.08; -0.71 ]	0.33 [0.1; 0.56 ]	0.57 [0.39; 0.74 ]	-1.80 [-1.98; -1.62 ]	0.60 [0.4; 0.79 ]	1.20 [1.03; 1.38 ]
65	with diabetes	3.23 [3.11; 3.34 ]	3.11 [2.87; 3.35 ]	-6.33 [-6.58; -6.09 ]	1.94 [1.82; 2.07 ]	3.91 [3.71; 4.1 ]	-5.85 [-6.08; -5.62 ]
65	with heart failure	-0.21 [-0.39; -0.04 ]	0.46 [0.24; 0.69 ]	-0.25 [-0.43; -0.07 ]	-1.13 [-1.31; -0.96 ]	0.73 [0.53; 0.92 ]	0.41 [0.22; 0.59 ]
65	with coronary heart disease	1.46 [1.28; 1.63 ]	0.5 [0.27; 0.73 ]	-1.96 [-2.15; -1.77 ]	0.57 [0.4; 0.74 ]	1.04 [0.84; 1.23 ]	-1.61 [-1.8; -1.42 ]
**age**	**disease**	**BMI < 25**	**25–30**	**30+**	**BMI < 25**	**25–30**	**30+**
75	entire population	-0.70 [-1.32; -0.08 ]	0.01 [-0.67; 0.7 ]	0.69 [0.25; 1.13 ]	-0.25 [-0.87; 0.37 ]	-0.08 [-0.77; 0.62 ]	0.33 [-0.25; 0.91 ]
75	with colorectal cancer	-0.57 [-0.76; -0.38 ]	0.00 [-0.23; 0.23 ]	0.56 [0.39; 0.74 ]	-0.96 [-1.15; -0.78 ]	0.13 [-0.1; 0.36 ]	0.84 [0.62; 1.05 ]
75	with breast cancer	-0.23 [-0.44; -0.03 ]	0.00 [-0.22; 0.23 ]	0.23 [0.08; 0.38 ]	2.58 [2.39; 2.77 ]	0.72 [0.49; 0.95 ]	-3.3 [-3.51; -3.09 ]
75	with cancer of the kidney	2.03 [1.84; 2.23 ]	-0.3 [-0.52; -0.07 ]	-1.73 [-1.89; -1.57 ]	1.85 [1.66; 2.05 ]	0.44 [0.21; 0.67 ]	-2.29 [-2.5; -2.09 ]
75	with endometrial cancer	-	-	-	-1.75 [-1.9; -1.59 ]	0.24 [0.01; 0.47 ]	1.51 [1.27; 1.74 ]
75	with cancer of the prostate	-0.23 [-0.44; -0.03 ]	0.00 [-0.22; 0.23 ]	0.23 [0.08; 0.38 ]	-	-	-
75	with stroke	1.25 [1.06; 1.44 ]	0.21 [-0.02; 0.44 ]	-1.46 [-1.63; -1.29 ]	0.81 [0.62; 1 ]	0.71 [0.49; 0.94 ]	-1.53 [-1.74; -1.32 ]
75	with diabetes	5.23 [5.09; 5.38 ]	1.6 [1.37; 1.83 ]	-6.83 [-7.05; -6.62 ]	4.44 [4.3; 4.57 ]	4.19 [3.96; 4.42 ]	-8.63 [-8.87; -8.39 ]
75	with heart failure	0.39 [0.2; 0.57 ]	0.45 [0.22; 0.68 ]	-0.84 [-1.02; -0.66 ]	-0.4 [-0.58; -0.22 ]	0.65 [0.42; 0.88 ]	-0.25 [-0.47; -0.03 ]
75	with coronary heart disease	2.28 [2.09; 2.46 ]	0.22 [-0.01; 0.44 ]	-2.49 [-2.67; -2.31 ]	1.36 [1.18; 1.54 ]	1.16 [0.93; 1.39 ]	-2.52 [-2.74; -2.31 ]
**age**	**disease**	**BMI < 25**	**25–30**	**30+**	**BMI < 25**	**25–30**	**30+**
85	entire population	-2.34 [-2.96; -1.71 ]	0.05 [-0.6; 0.69 ]	2.29 [1.91; 2.67 ]	-1.2 [-1.81; -0.58 ]	-0.71 [-1.39; -0.03 ]	1.90 [1.31; 2.5 ]
85	with colorectal cancer	-1.75 [-1.94; -1.55 ]	0.18 [-0.04; 0.39 ]	1.57 [1.42; 1.72 ]	-1.16 [-1.34; -0.98 ]	-0.07 [-0.3; 0.15 ]	1.23 [1.01; 1.45 ]
85	with breast cancer	-0.78 [-0.99; -0.57 ]	0.02 [-0.2; 0.23 ]	0.76 [0.64; 0.89 ]	1.91 [1.71; 2.1 ]	0.37 [0.15; 0.6 ]	-2.28 [-2.49; -2.08 ]
85	with cancer of the kidney	0.83 [0.62; 1.03 ]	-0.37 [-0.58; -0.15 ]	-0.46 [-0.59; -0.33 ]	0.83 [0.64; 1.03 ]	0.09 [-0.14; 0.31 ]	-0.92 [-1.12; -0.72 ]
85	with endometrial cancer	-	-	-	-1.96 [-2.11; -1.8 ]	-0.39 [-0.61; -0.16 ]	2.34 [2.11; 2.57 ]
85	with cancer of the prostate	-0.78 [-0.99; -0.57 ]	0.02 [-0.2; 0.23 ]	0.76 [0.64; 0.89 ]	-	-	-
85	with stroke	1.67 [1.47; 1.87 ]	-0.33 [-0.54; -0.11 ]	-1.34 [-1.48; -1.2 ]	1.86 [1.67; 2.05 ]	0.56 [0.34; 0.79 ]	-2.42 [-2.63; -2.22 ]
85	with diabetes	7.14 [6.96; 7.31 ]	-0.53 [-0.74; -0.32 ]	-6.61 [-6.79; -6.43 ]	7.44 [7.29; 7.59 ]	3.56 [3.34; 3.78 ]	-11.00 [-11.23; -10.77 ]
85	with heart failure	0.67 [0.48; 0.86 ]	0.12 [-0.09; 0.34 ]	-0.80 [-0.96; -0.63 ]	0.63 [0.45; 0.8 ]	0.68 [0.46; 0.91 ]	-1.31 [-1.53; -1.09 ]
85	with coronary heart disease	2.64 [2.45; 2.84 ]	-0.49 [-0.7; -0.27 ]	-2.16 [-2.3; -2.01 ]	2.62 [2.43; 2.8 ]	0.97 [0.75; 1.19 ]	-3.59 [-3.8; -3.37 ]

Here, the largest effects are seen for diabetes, where the prevalence in the newborn cohort builds up slower with age in comparison to the initial population. One explanation is that BMI increases with age, and as a result the higher categories of BMI also contain recent entrants, with a lower prevalence of disease. The difference between the two results can be as large as 11 percent points. For the other diseases, with smaller relative risks, differences stay below 4 percent points.

## Discussion

Our simulations show that, in general, the approximation method for constructing an initial population from marginal data implemented in DYNAMO-HIA clearly outperforms assuming independence. In many situations it performs reasonably well, but in some the proportion of the risk-factor is under- or overestimated by as much as 10 percentage points. For determining comorbidity between diseases that are linked by a common causal disease or common risk-factor the method also performs reasonably well. The method performs poorly in determining the comorbidity between a causal disease and the disease it causes.

In this paper, we compared joint distributions between a method for constructing an initial population and results of a simulation model. Both the model and the method largely make the same assumptions; for instance, they use the same relative risks between smoking (or BMI) and diseases, and between diabetes and cardiovascular diseases. Our work tests the consistency of the method of population construction within this simulation context. We are not aware of other papers describing similar validation exercises, although we do expect that similar exercises have been carried out as part of model construction elsewhere.

We showed that the approximation method performs poorly in determining the comorbidity between a causal disease and the disease it causes: It ignores the probability that the causal disease occurs second. Essentially, our approximate method assumes, for instance, that the relative risk of diabetes on CHD applies to all subjects with both diabetes and CHD. However, in some of these subjects CHD occurred first and diabetes second, not influenced by the extra risk of diabetes on CHD. Still, it would be easy to adapt the method to take this into account. One solution would be not to use the RR of diabetes on CHD but rather a weighted average of this RR and 1, where the weight would be determined by the ratio of the incidence rates of both diseases.

The reason that the approximate method deviates from the simulation is that prevalence rates of diseases result from incidence and disease-related excess mortality at earlier times. Initial population construction assumes that the instantaneous relative risk at a certain age applies also to earlier ages. As relative risks change with age, this is no longer the case. In our simulations we looked at the magnitude of this effect in practice. For COPD, and for lung and oral cavity cancer, the relative risks of smoking increase with age in the earlier period of life. For these diseases we therefore expect the approximate method to overestimate disease prevalence in smokers at a certain age (and, by extension, the number of smokers in those with the disease), because the prevalent cases stem partly from an earlier age, when lower relative risk applied. Indeed, this is what we observed. However, this effect is also seen in other diseases, where the relative risks remain constant or even slightly decrease with age between birth and late middle age. An explanation might be that in our data the proportion of never smokers decreases with age—for men nearly until age 60, and for women for all ages. This has implications for the net transition rates. In order to keep the age-specific prevalence of never smokers constant, smoking initiation in the simulation should continue at a fairly high rate, almost until age 60 for men, while doing so continuously for women. This means that the smokers in the simulation contain a fair amount of recent smokers, who have a lower prevalence of disease than long-term smokers. This the reason we see a lower prevalence of smoking in individuals with almost any disease. The only exception is diabetes, where no relation between smoking and diabetes was assumed. This deviation is therefore artificially induced by using net transition rates. These rates are not realistic, because smoking initiation over the age of 20 is actually quite rare. Smoking is a kind of worst case scenario, since smoking behavior differs greatly between generations. The underlying assumption (that the past age- and sex-related smoking rates were equal to the current ones) is therefore is unrealistic as well. Only simulating generations separately would yield a more realistic picture. However, this kind of simulation would require historical data on smoking rates, which are not readily available in the detailed form we need. Instead, we chose to run a second simulation using BMI. Although generational effects are also present for BMI, this effect is both smoother, and smaller than the physiological effects of aging.

The case of BMI is similar: those in higher BMI categories are a mix of individuals entering this category at different times. Generally, the more recent the entrance, the lower the prevalence. For BMI, this process is more realistic. For diabetes, where relative risks of BMI are also fairly high and decline with age, this, too, causes the approximate method to deliver inaccurate prevalence rates of diabetes. Most other diseases show lower relative risks, and therefore also showed less deviance of the approximate method for the full simulation of a new-born cohort.

There is no hard-and-fast rule for how much deviation of the approximate population construction method we should accept. In generating simulation results, comorbidity only plays a minor role, mostly because it is involved in estimating disease-attributable mortality from disease related excess mortality. Thus, only second-order effects of differences in comorbidity ratios on simulation results are expected. The joint distribution of the risk-factor and the disease has a similar role in estimating disease-attributable mortality, and it further influences simulation results insofar as a higher prevalence of disease depletes the population of individuals still liable to get sick. This will result in incidence rates that are distributed slightly differently for those with and without the risk-factor. This phenomenon will also have second-order effects on the results of a simulation study. Since smoking represents a worse-case scenario, our work indicates that the approximate method, though imperfect, will work well enough in most situations.

The validation method proposed here for constructing an initial population might also turn out useful for situations where the initial population is based on microdata. Here, considerable differences between the projections based on a newborn cohort and the initial population–in the absence of important changes in disease dynamics in the recent past—could give clues as to which update rules in the simulation might require tuning. Our method checks the consistency between the initial population and the simulation model that uses it. Such consistency is necessary but not sufficient for validating a model, as the simulation model might itself be invalid. Nevertheless, we believe that applying a procedure such as ours is a necessary step before using any model.

An alternative method for initiating a simulation would be to use the simulation model to simulate subjects from birth to the simulation's starting point[2]. However, our simulations with net-transition rates for smoking also show that only reliable information on the past history of risk-factor exposure can ensure accurate results. This information is often hard to come by. Additionally, this method would require calibration of historic incidence, mortality and risk-factor transition rates in order to deliver correct marginal proportions of the risk-factor as well as marginal prevalence rates of each disease in the initial population. With many diseases in a model this requires serious efforts.

We conclude that although imperfect, the approximate method for construction an initial population used in the DYNAMO-HIA model work considerably better than any assumption of independence between risk-factor and disease occurrence. However, the method for determining the amount of comorbidity between diseases that are causally linked can be further improved. Given the existing discrepancies, however, developing more elaborate methods based on running a simulation model could be worthwhile for some situations.

## Appendix A: Construction of the initial population in DYNAMO-HIA

DYNAMO-HIA uses the following algorithm to simulate the initial population (adapted from Boshuizen et al. 2012 [1]):

In the case of diseases that only depend on the risk-factor state, r (henceforth known as “independent diseases”), we solve the baseline odds of disease i iteratively from

$$\mathrm{Pr}\left(d_{i}=1\right)=\sum_{r}\frac{{RR}_{r\to d_{i}\;{Baseline\;odds}_{i}}}{{RR}_{r\to d_{i}}\;{Baseline\;odds}_{i}+1}Pr(R=r)$$ (A1)

where Pr(di = 1) is the (marginal) prevalence of disease i (input to the model); Pr(R = r) is the (marginal) probability of the risk-factor state r (input to the model); and ${RR}_{r\to d_{i}}$ is the relative risk of risk-factor state r on the incidence of disease i (input to the model).

With these baseline odds, we can then calculate the joint prevalence of risk-factor states and independent diseases, using the relative risks (assuming them to be equal to the POR). If we then define a new risk-factor state, r*, as the joint risk-factor–independent disease state, we can repeat the procedure for diseases that depend on other diseases (dependent diseases) using r*. Although in theory the process could be repeated, adding extra layers of new dependent diseases in each step, the current implementation of the DYNAMO-HIA model is restricted to a single layer of dependent diseases.

Next, based on a joint distribution of all risk-factor and disease states, the model generates an initial population for the simulation. The first step is to assign risk-factor states until the number of simulated individuals assigned nearly (or completely) reaches the expected number under the intended distribution; subsequently, the remaining simulated persons are randomly assigned a risk-factor value. Given the assigned risk-factor, the disease state is then calculated based on Eq (A1).

## Appendix B: Update rules in DYNAMO-HIA

DYNAMO-HIA applies an epidemiological model that defines transition rates from being without a disease to having the disease (incidence rate) and from being alive to death (mortality rate). The epidemiological model of DYNAMO-HIA parameterizes two update rules: one using incidence rates and one using mortality rates. These are described below (adapted from Boshuizen et al. 2012 [1]):

### Incidence rates

The transition rate from not having disease i to having disease i is called the (nonfatal) incidence rate of i and is described by Eq (B1):

$$I_{i}(r,C)-I_{0,i}{RR}_{r\to d_{i}}\prod_{c_{j}=c_{1}}^{c_{j}=c_{m}}{RR}_{c_{j}\to d_{i}},$$ (B1)

where r is a risk-factor state; C is a vector of states of the causal diseases—that is, diseases that are a cause of another disease) with elements {c1, …, cm} (e.g., for five causal diseases: {0,0,1,0,0}); I0,i is the baseline incidence of disease i—that is, the incidence in the state where all relative risks are equal to 1; ${RR}_{r\to d_{i}}$ is the relative risk of risk-factor state r on disease i; and ${RR}_{r\to d_{i}}$ is the relative risk of causal disease j on disease i.

In Eq (B1), the baseline incidence I0,i is estimated from the input data on population incidence (specified by age and sex), while the relative risks (also specified by age and sex) are data input to the program. The program automatically calibrates the baseline incidence in order to make the first year incidence equal to the input population incidence.

### Mortality rate

DYNAMO-HIA contains three different disease processes determining mortality: (1) a chronic disease process characterized by a constant (attributable) mortality rate after the individual gets a disease that is only age- and sex-dependent (in our case, COPD, diabetes and congestive heart failure); (2) a disease process in which the disease can be acutely fatal (in our case, CHD and stroke); (3) a disease process that includes a “cured” fraction, which was applied to all cancers.

For diseases with a constant mortality rate, the transition rate to death given risk-factor state r and disease state D [the mortality rate M(r,D)] is given by E

$$M(r,D)=M_{0,OC}{RR}_{r\to OC}+\prod_{i=1}^{i=n}{Am}_{i}d_{i},$$ (B2)

where M(r,**D**) is the mortality rate given risk-factor state r and disease state D; r is the risk-factor state; D is a vector of states of diseases with elements {d1, …, dn}; Ami is a model parameter referred to as attributable mortality, with the restriction that the mortality rate is due exclusively to disease i; *RR*_*r*→*OC*_ is the relative risk of risk state r for other causes of mortality (i.e., other than from the diseases included in the model); and *M*_0,*OC*_ is the baseline other causes of mortality.

The dependence of the other-cause mortality on risk-factor status is optional in the model. Without this dependence, the other-cause mortality is the same for all simulated subjects, and the risk-factor has only an indirect effect on mortality: namely, through its effect on disease incidence.

For diseases that can be acutely fatal, a term is added to the mortality rate M(r,D) given by Eq (B2), as follows:

$${M(r,D)=M}_{0,OC}{RR}_{r\to OC}+\sum_{i=1}^{i=n}{Am}_{i}+\sum_{i=1}^{i=n}{CF}_{i}(r,D)$$ (B3)

where *CF*_*i*_(*r*,**D**)

$${CF}_{i}(r,D)=M_{0,{CF}_{i}}{RR}_{r\to d_{i}}\prod_{c_{j}=c_{1}}^{c_{j}=c_{m}}{RR}_{c_{j}\to d_{i}},$$ (B4)

where cj is an element of the vector of causal disease states C; $M_{0,{CF}_{i}}$ is the baseline fatality rate of disease i; ${RR}_{r\to d_{i}}$ is the relative risk of risk-factor state r for disease i; and ${RR}_{r\to d_{i}}$ is the relative risk of causal disease j for disease i.

A disease with a cured fraction is basically split up at the time of diagnosis: a “cured disease” and a “not-cured disease.” Individuals with the latter have an increased mortality rate attributable to the disease (attributable mortality), which is constant over time, while those with the former have an attributable mortality of 0.

For both the cured and not-cured disease, M(r,**D**) is given by Eq (B2) setting Ami to 0 for the cured disease.

## Supporting information

S1 Fig Relative risks used in the simulation for smoking. Dark blue: current smokers, men; light blue: former smokers, men; red: current smokers, women; pink: former smokers, women. If only red and pink lines are present, the same relative risks have been used for men and women.
(TIF)

S2 Fig Relative risks used in the simulation for BMI. Dark blue: BMI 30 or more, men; light blue: BMI 25–30, men; red: BMI 30 or more, women; pink: BMI 25–30, women. If only red and pink lines are present, the same relative risks have been used for men and women.
(TIF)

S3 Fig Relative risks used in the simulation for diabetes. Blue: men; red: women. When only a red line is visible, identical RRs have been used for men and women.
(TIF)
