# Supplementary figures and images for: Internal consistency of a synthetic population construction method for chronic disease micro-simulation models
Source: PLoS One. 2018 Nov 15;13(11):e0205225. doi: 10.1371/journal.pone.0205225 (PMC6237328; doi:10.1371/journal.pone.0205225)

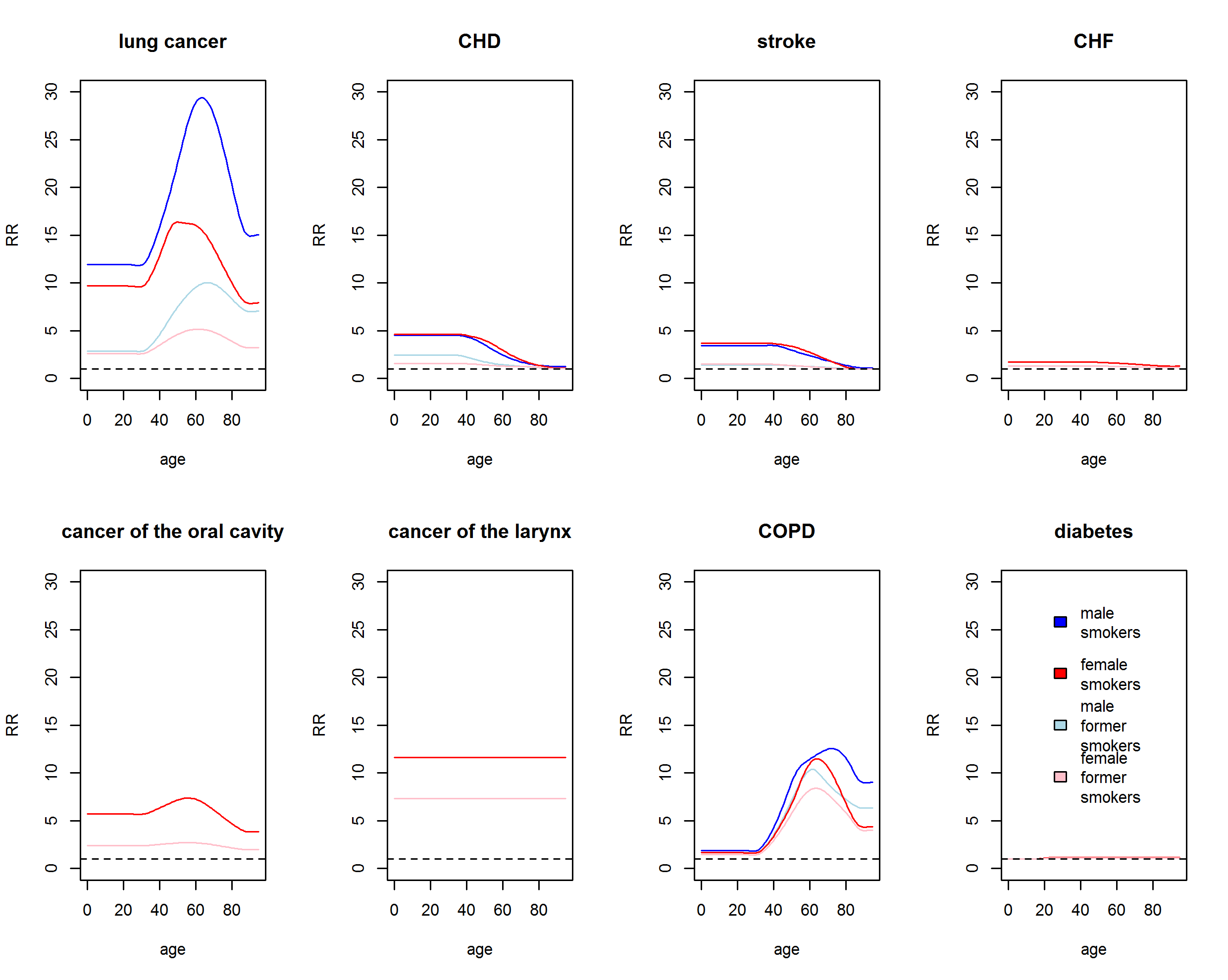

Supplement: S1 Fig — Dark blue: current smokers, men; light blue: former smokers, men; red: current smokers, women; pink: former smokers, women. If only red and pink lines are present, the same relative risks have been used for men and women. (TIF) [file pone.0205225.s001.tif]

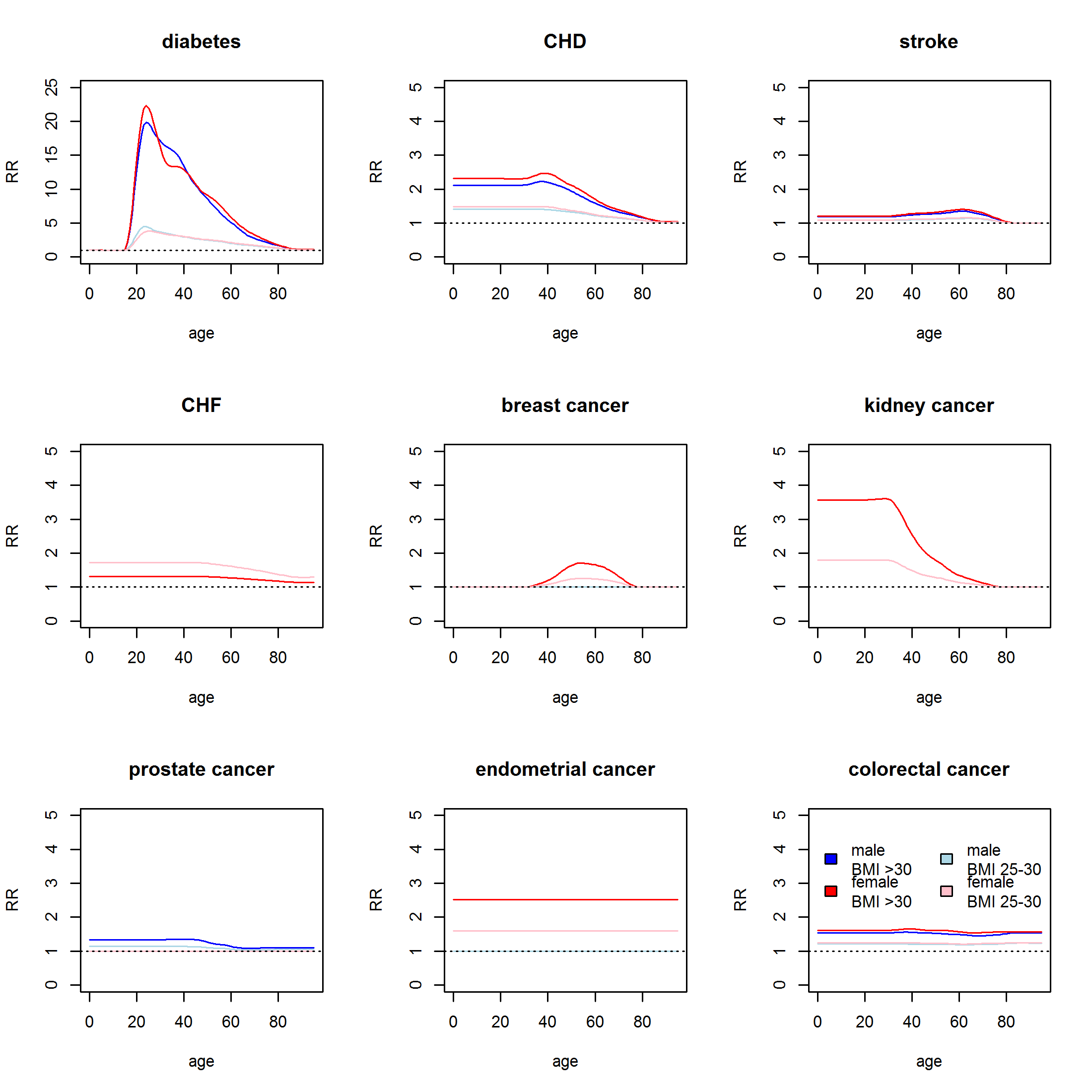

Supplement: S2 Fig — Dark blue: BMI 30 or more, men; light blue: BMI 25–30, men; red: BMI 30 or more, women; pink: BMI 25–30, women. If only red and pink lines are present, the same relative risks have been used for men and women. (TIF) [file pone.0205225.s002.tif]

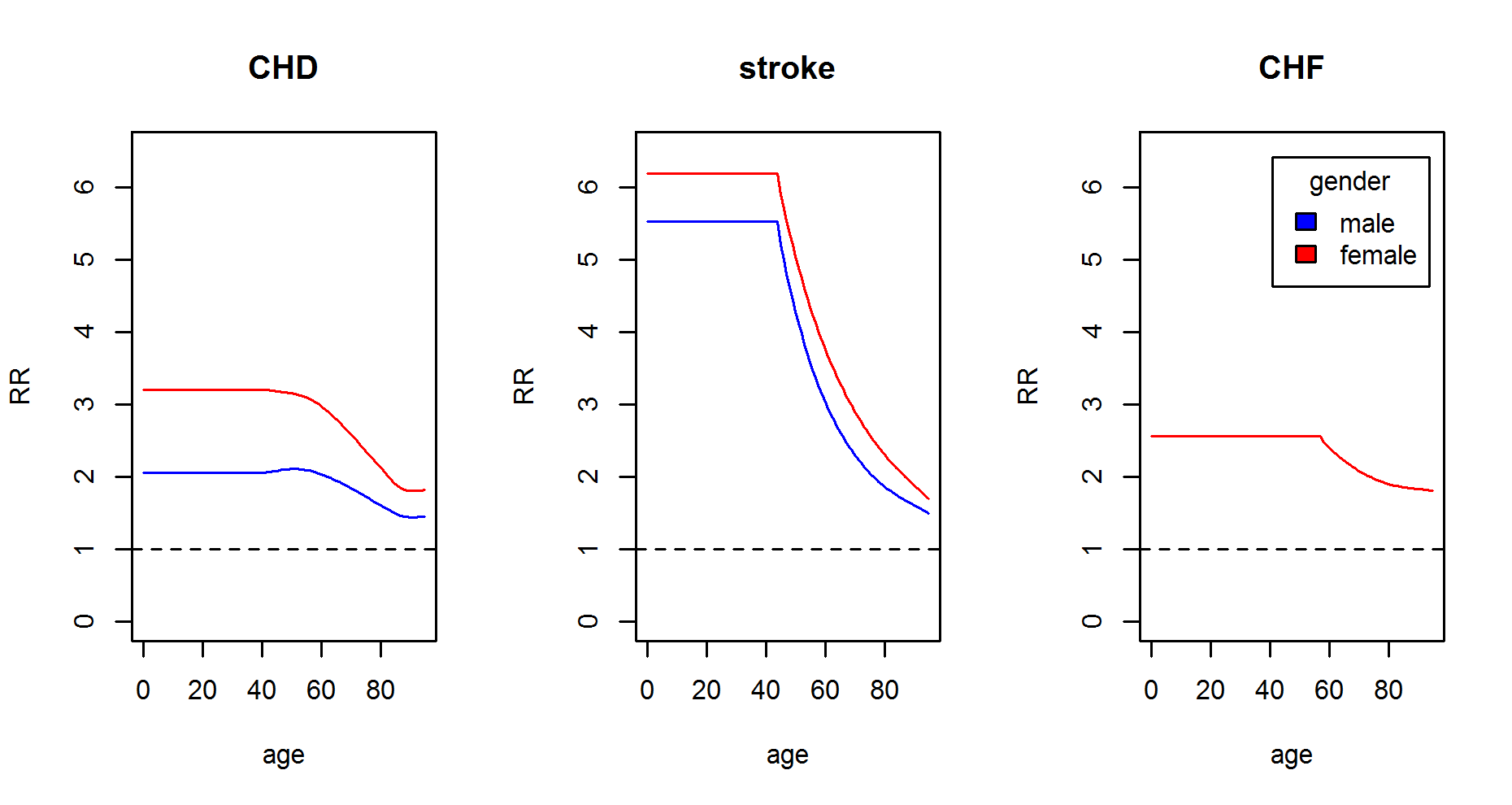

Supplement: S3 Fig — Blue: men; red: women. When only a red line is visible, identical RRs have been used for men and women. (TIF) [file pone.0205225.s003.tif]
